# Characterization of Oral Squamous Cell Carcinoma Associated Inflammation: A Pilot Study

**DOI:** 10.3389/froh.2021.740469

**Published:** 2021-09-21

**Authors:** Catherine Laliberté, Nicole Ng, Denise Eymael, Kevin Higgins, Aiman Ali, Alex Kiss, Grace Bradley, Marco A. O. Magalhaes

**Affiliations:** ^1^Oral Pathology and Oral Medicine, Faculty of Dentistry, University of Toronto, Toronto, ON, Canada; ^2^Cancer Invasion and Metastasis Laboratory, Faculty of Dentistry, University of Toronto, Toronto, ON, Canada; ^3^Department of Otolaryngology-Head and Neck Surgery, Odette Cancer Centre, Sunnybrook Health Sciences Centre, Toronto, ON, Canada; ^4^Evaluative Clinical Sciences, Sunnybrook Research Institute, Sunnybrook Health Sciences Centre, Toronto, ON, Canada; ^5^Dental and Maxillofacial Sciences Department, Sunnybrook Health Sciences Centre, Toronto, ON, Canada

**Keywords:** oral cancer, inflammation, dysplasia, saliva, cancer, cytokine, chemokine, IL6

## Abstract

**Background:** Oral squamous cell carcinoma (OSCC) is a devastating disease that is usually associated with a dense associated inflammatory infiltrate. Characterizing tumor-associated inflammation is critical to understand the pathogenies of tumor development and progression.

**Methods:** We have tested a protocol to analyze tissue and salivary immune cells and mediators of 37 patients with OSCC at different stages and compared to eight chronic periodontitis patients and 24 healthy controls. Tissue analysis was based on fluorescent immunohistochemistry (FIHC) and inflammatory mediators were analyzed using a Luminex-based 30-Plex panel. Immune cells were analyzed using multichannel flow cytometry including CD45, CD66b, CD3, CD4, CD8, CD25, CD56, CD68, CD138, PD-1, and PD-L1.

**Results:** We show an increase in OSCC-associated inflammation characterized by increased pro-inflammatory cytokines including IL-6, IL-8, TNFα, and GMCSF and increased salivary immune cells.

**Conclusion:** We described a new method to analyze salivary inflammatory markers that can be used in future studies to monitor disease progression and prognosis.

## Introduction

Oral squamous cell carcinoma (OSCC) is a neoplasm with squamous differentiation arising from the mucosal epithelium of the oral cavity [1] and accounts for 75–90% of malignant tumors in this anatomical location [1, 2]. It is associated with significant morbidity and mortality because of the importance of oral tissues in chewing, swallowing, speaking, and facial appearances [3]. The annual world-wide age-standardized incidence rate of OSCC in 2018 was 5.8 cases for males and 2.3 cases for females per 100,000 population per year, with a global mortality rate of 2.8 deaths for males and 1.2 deaths for females per 100,000 per year [4]. The prognosis for OSCC has not improved greatly over the last decades, as the 5-year survival rate is around 60% [5]. The poor outcome of OSCC is mostly due to late diagnosis usually made once the cancer has reached advanced stages [3, 5]. Thus, understanding the pathogenesis of OSCC is critical for early detection and to improve clinical outcomes.

Part of the challenges in understanding oral squamous cell carcinoma progression is the unique environment of the oral cavity which has well-developed innate and adaptive immune responses, frequent contact with the external environment, and exposure to carcinogenic factors along with a diverse microbiome [6]. In this context, oral keratinocytes interact with immune cells and mediators that have been linked to the pathogenesis and progression of cancers, including increased rates of malignant transformation of oral lesions [7–9]. Oral squamous cell carcinoma usually presents with significant associated inflammatory response on histopathological examination but the relationship between inflammation and OSCC progression is still poorly understood. We have previously found a five-fold increase in neutrophils and T-cells in tissue samples and a 10-fold increase in pro-inflammatory cytokines, particularly TNFα in saliva of OSCC patients compared to healthy controls [10, 11]. Our previous results established that TNFα promotes invasion in oral keratinocytes and increases expression of cytokines, which in turn recruits immune cells that express more TNFα, thereby creating a paracrine signaling pathway [10, 11].

Several studies have evaluated blood and saliva cytokines in head and neck squamous cell carcinomas. Recent studies found different salivary levels of some cytokines among oral cancer, OPMD, and healthy patients, suggesting these cytokines as potential diagnostic biomarkers for oral cancer and OPMD [12]. Rhodus et al. studied levels of proinflammatory, angiogenic, NF-kB dependant cytokines IL-1, IL-6, IL-8, and TNF-a in whole stimulated saliva and showed that these were significantly elevated in OSCC patients compared to subjects with oral premalignant disorders (OPMD) and controls [13]. Using ELISA, Korostoff et al. investigated salivary levels of IL-1a, IL-6, IL-8, VEGF-a, and TNFα and found that all five cytokines were elevated in conventional tongue OSCC group compared to control groups [14]. Similar increases in pro-inflammatory cytokine IL-6 in OSCC patients were also reported by Katakura et al. and Sato et al. [15, 16]. Salivary IL-6 concentration was significantly higher in patients with locoregional recurrence at 24 months than patients without recurrence, and IL-6 was an independent risk factor for locoregional recurrence. Arellano et al. confirmed increased IL-1β and IL-8 in a group of OSCC patients compared to control patients by ELISA and Luminex technology, validating the use of the latter as a reliable method for quantification of salivary proteins [17]. Brinkman determined that salivary levels of IL-8 and IL-1β are elevated in OSCC patients [18], while Lee et al. found significant difference in GCSF, IL-6, and eotaxin between early and late OSCC [19]. Similar results were seen in the peripheral blood of patients with HNSCC where IL-6, IL-8, and VEGF were present at higher concentrations in the blood of patients compared to healthy control subjects [20]. In parallel, IL-1β, IL-6, and TNFα were found to be higher in OSCC patients [21].

Cancer patients often have defective antitumor immunological responses [7]; despite a dense inflammatory infiltrate often seen in tumor tissue, efficient antitumor response is not present, and rather represent an aberrant host inflammatory response. The expression of various immune mediators and the abundance of different cells in tumor microenvironment can determine whether the immune balance is tipped toward tumor-promoting inflammation or antitumor immunity [22, 23].

In this study, we established a protocol for comprehensive analysis of the inflammatory response to OSCC in saliva and tissue samples, using both cytokines and immune cells compared to saliva of patients with a chronic oral inflammatory disease and healthy controls. We used the protocol to provide evidence that OSCC is associated with distinctive inflammatory response that could be observed in tissue and salivary cytokines.

## Materials and Methods

### Study Design

This was a prospective, case-controlled pilot study to investigate inflammatory changes in saliva in patients with OSCC. The study has been approved by the Research Ethics Board (REB) of Sunnybrook Health Sciences Center (project identification number 223-2015) and University of Toronto (protocol number 32724).

### Participants

The study population consisted of all patients referred for treatment of biopsy-proven OSCC at the Odette Cancer Center, Sunnybrook Health Sciences Center, Toronto, between September 2016 and October 2017. A total of 37 patients consented to participate in this study. A control group of 24 healthy patients with no history of oral cancer, as well as eight patients from the University of Toronto Faculty of Dentistry with moderate to severe periodontal disease and no history of oral cancer were included. The control patients were matched in age and sex with the OSCC patients. Table 1 shows the demographic information of the study and control populations.

**Table 1:** Age and sex distribution among cases.

		**Control (24)**	**Perio (8)**	**Dysplasia (5)**	**OSCC (32)**
Age	Mean	61 ± 14	51 ± 14	70 ± 16	66 ± 13
Sex	Male	9 (37.50%)	4 (50%)	1 (20%)	14 (43.8%)
	Female	15 (62.5%)	4 (50%)	4 (80%)	18 (56.3%)

*Age and sex distribution in the individual groups*.

### Inclusion and Exclusion Criteria

The patients were recruited into four groups: (1) control patients without oral or systemic diseases, (2) Patients with a diagnosis of OSCC, (3) Patients with periodontal disease, and (4) Patients with dysplasia at resection. *OSCC group*: Inclusion criteria: biopsy proven OSCC referred for treatment at the Odette Cancer Center. Exclusion criteria: tumor location outside the oral cavity, non-squamous cell carcinoma, history of previous malignant disease, chemotherapy or head and neck radiation within the previous 5 years, presence of a second primary malignancy, immune disorders (such as Sjogren's syndrome and HIV infection), history of hepatitis, and severe systemic disease (ASA > 4). Patients with a biopsy confirmed OSCC that presented for surgery at the Odette cancer center but were found to have only premalignant lesions (dysplasia) and no invasive OSCC were included in the *dysplasia group*.

*Control groups*: healthy control patients with no reported oral disease or ongoing dental/periodontal treatment were included. *Periodontal disease group*: Periodontal disease patients from the Periodontal Disease Unit at the University of Toronto Faculty of Dentistry were only included if they had treatment appointments scheduled for diagnosis of moderate to severe periodontal disease, as recorded in their dental chart. Exclusion criteria included: history of previous malignant disease, chemotherapy or head and neck radiation within the previous 5 years, presence of a second primary malignancy, immune disorders (such as Sjogren's syndrome and HIV infection), history of hepatitis, and severe systemic disease (ASA > 4).

### Protocol for Collection of Saliva Samples

As a pilot project, our first objective was to test a protocol for collection and analysis of saliva samples. The collection protocol using 3 ml of a saline rinse was well-tolerated by all patients and all patients consented to the collection of saliva after the study and protocols were explained. The collection was carried during the pre-surgical consult and did not cause any disruption to standard treatment protocols. The filtered saliva supernatant was adequate for cytokine analysis as all samples passed initial quality control and were within the range of the Luminex test. The establishment of FMO and single stain controls required the use of a control saliva sample as blood controls showed different staining patterns compared to the saliva samples. To overcome this issue, a single donor collected multiple saliva samples that were combined and used to generate all FMO and single marker controls.

**Figure 1:**
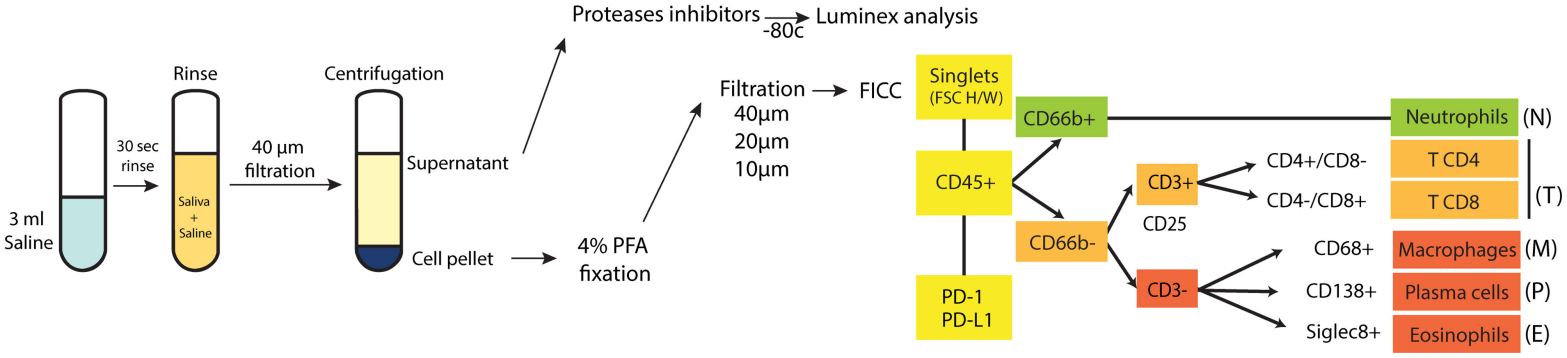
Study protocol. A diagram showing the protocol for collecting and analyzing saliva samples, with the gating strategy used to identify and quantitate each inflammatory cell type.

All patients were recruited during a pre-treatment oncology appointment at the Odette Cancer Center, Sunnybrook Hospital, Toronto. After informed consent was obtained, a short medical questionnaire and intraoral examination were performed. Saliva samples were obtained by rinsing the mouth for 30 s with 3 ml of saline. The samples were kept on ice and stabilized with 100 μl of protease inhibitor (EMD Millipore Protease Inhibitor Cocktail Set II, Calbiochem) until filtration using a 40 μm membrane filter. Time interval between collection and filtration of samples ranged from 20 to 150 min. The saliva samples after filtration were centrifuged (7 min at 3,000 rpm) in the Sunnybrook Hospital Clinical Pathology lab to obtain cell pellets and supernatants. The latter were frozen at −80°C until use. Cell pellets were re-suspended and fixed in 200 μl paraformaldehyde 4% and PBS and kept at 4°C until use for a duration ranging from 1 to 12 months (Figure 1). Control subjects and subjects with periodontitis provided saliva samples that were processed at the Faculty of Dentistry, University of Toronto. Inflammatory cell counts in blood were obtained from routine pre-surgical blood tests at the Odette Cancer Center, without change to standard of care or extra visits.

### Inflammatory Cell Analysis by Multichannel Flow Cytometry

A 12-antibody panel (Supplemental Table 1) was optimized to determine the inflammatory cell profile in saliva. Prior to staining the cells, all samples were re-filtered with a 40 μm, 20 μm membrane filter, and then with a 10 μm membrane filter to remove debris. The cells were washed with PBS, and concentration was adjusted to a cell number of 1–5 × 10^6^ cells/ml with 300 μl ice cold FACS buffer. Two Hundred microliters of Fc receptor blocking solution (5 μl of TruStain FcXTM per million cells) was added to each sample (Fc blocking solution 1% BSA and 1% FBS diluted in PBS buffer) and incubated on ice for 10 min. Samples were centrifuged at 1,500 rpm for 5 min at 4°C. Supernatant were discarded. Primary labeled antibodies were added and incubated for 20 min at room temperature in the dark. Cells were washed three times by centrifugation at 1,500 rpm for 5 min and resuspended in 1,000 μl of ice cold FACS buffer and kept in the dark at 4°C until analysis. Vortex and a final filtration (10 μm filter) were done immediately before FCM to ensure even distribution of the cells in the samples and to minimize cells and particles aggregates.

Fluorescent-labeled saliva samples from the study and control patients (total 69 samples) were analyzed in a flow cytometer (BD Fortessa x20 5 laser) in three sessions. For each FCM session, the instrument was calibrated with rainbow beads (BD Bioscience), to ensure that the voltage applied to lasers/detectors always returned the same median fluorescence. Compensation beads stained with the 12 fluorescent antibodies were used to set voltage and adjust compensation matrix. Full minus one controls (FMOs) for each of the 12 stains, as well as one fully stained control and one unstained control were prepared, using saliva provided by a single healthy donor. The instruments were calibrated with the same parameters for each session. Supplemental Table 1 shows the laser configuration and antibodies/fluorophore used for detection of immune cells. For 38 of the 69 samples, 100,000 events were analyzed. For 30 of the 69 samples (10 cancer samples, seven perio samples, 13 healthy samples) the entire sample was analyzed in order to calculate the total number of immune cells in the samples. FlowJo 10.4.1 software was used for data analysis. Parameters that were studied included forward scatter (to analyze cell size) and side scatter (to analyze cell granularity and complexity), followed by fluorescent emission analysis of the 12 selected fluorescence-tagged antibodies (to sort immune cells subsets based on specific cell surface fluorescent-labeled antigens). Figure 1 shows the gating strategy that was developed to identify and quantitate each inflammatory cell type.

### Cytokines Analysis

Assays were conducted in 96-well plates according to the immunoassay protocol for the Millipore Human Cytokine/Chemokine Magnetic Bead Assay Panel (HCYTMAG-60K-PX30 by EMD Millipore, USA) [including analytes EGF, G-CSF, GM-CSF, IFN-α2, IFN-γ, IL-1α, IL-1β, IL-1ra, IL-2, IL-3, IL-4, IL-5, IL-6, IL-7, IL-8, IL-10, IL-12 (p40), IL-12 (p70), IL-13, IL-15, IL-17, IP-10, MCP-1, MIP-1α, MIP-1β, TNF-α, TNF-β, VEGF, RANTES/CCL5, Eotaxin/CCL11] and Luminex? detection. The samples were prepared, and the data acquired and analyzed at the Princess Margaret Genomic Center (PMCC, Toronto, Canada) according to the manufacturer's instructions. Briefly, 25 μl magnetic beads, 25 μl assay buffer, and 25 μl of sample (1:2 diluted sample) were incubated overnight at 4°C with shaking. Beads were subsequently washed 2 X times and incubated with 25 μl of detection antibody for 1 h. Twenty-five microliters streptavidin phycoerythrin was added to the assay mixture for 30 min at room temperature. Beads were washed 2X and resuspended in 150 μl of Sheath Fluid. Assays were read with Luminex 100 Reader and data was analyzed using Bio plex Manager 6.0. Analyte concentrations are indicated in pg/ml units. From the 69 patients included in this cytokine analysis, 30 samples (17 cancer patients and 13 controls) were used as part of a previous publication [11].

### Tissue Analysis

A total of 25 cancer cases were available for analysis and 10–15 tissue slides were prepared from the resection specimens. Fluorescent immunohistochemistry (FIHC) was performed as described previously [11]. In brief, primary antibodies for PD-1, PD-L1, CD45, CD66b, CD3, CD4, and CD8 surface markers were used to assess the populations of neutrophils (CD45+ CD66b+), CD8+ T cells (CD3+CD8+), and CD4+ T cells (CD3+CD4+) in addition to PD-1 and PD-L1 distribution based on the colocalization of the two markers. Using Volocity software, all tissue samples were analyzed, and the average sum of pixel intensity was calculated for each slide.

### Statistical Analysis

Descriptive statistics, including means and standard deviations, were calculated for the outcome variables. These statistics were reported for the whole sample as well as separately by the four groups of interest. The Shapiro test of normality was run on the outcome variables and following this, non-parametric Kruskal-Wallis tests were used to examine whether there are significant bivariate associations between group membership and potential predictor variables. To explore differences among groups, multivariable logistic regression was applied to examine the relationship between group membership and predictors of interest. In the logistic regression model, group membership was dichotomized as Cancer vs. the others. Prior to modeling, multicollinearity among the predictors was examined using tolerance statistics (tolerance value <0.4). If multicollinearity was found to exist, then only one member of a correlated set of variables was retained for the final model. The model results were reported as odds ratios and their associated 95% confidence intervals. All analyses were carried out using SAS 9.4 (Cary, NC, USA). Correlation analyses and graphs were generated using GraphPad Prism 7.0 and IBM SPSS version 25. All figures were prepared using Adobe Illustrator CC 2019.

## Results

### Patient Characteristics

The demographics of the groups can be seen in Table 1. Twenty-eight [24] patients were treated with surgery and their tumors staged according to AJCC 7^th^ ed. based on the pathology report of the tumor resection; clinical staging was only used for two patients treated with primary chemoradiation and two patients did not have definitive treatment at the Odette Cancer Center (Table 2). Five patients had an initial biopsy of dysplasia suspicious for carcinoma, and showed only dysplasia (moderate to severe) in the resection specimen. These were included in the dysplasia group. There were no significant differences in the age distribution between control (61 ± 14), periodontal disease (51 ± 14), dysplasia (70 ± 13), or cancer patients (66 ± 13) (*P* = 0.07). There were more females in all groups except periodontal disease group (*P* = 0.049). Further analysis of dysplasia and cancer patients reveal that the most commonly involved site in all groups was tongue (*n* = 19, 51.3%) followed by gingiva (*n* = 9, 24.3%). 86.4% of lesions were painful and 40% of patients had abundant visible dental plaque. The presence of visible plaque and daily tooth brushing was significantly increased in periodontal disease patients compared to cancer patients (*P* = 0.0498 and *P* = 0.0333). There were no significant differences in alcohol consumption or smoking between groups and between cancer stages. Most dysplasia and cancer patients had a normal appearing remaining oral mucosa (45.9%, *n* = 17/37) while 18.9% (7/37) had other areas of leukoplakia (Table 2).

**Table 2:** Clinical features of the lesions.

		**Dysplasia**	**Stage I**	**Stage II**	**Stage III**	**Stage IV**
**Patients (** ***n*** **)**		**5**	**10**	**5**	**4**	**13**
Location	Tongue	3 (60%)	6 (60%)	2 (40%)	2 (50%)	6 (46.2%)
	FOM	0	2 (20%)	0	0	1 (7.7%)
	Buccal Mucosa	0	0	2 (40%)	1 (25%)	2 (15.4%)
	Gingiva	2 (40%)	1 (10%)	1 (20%)	1 (25%)	4 (30.8%)
	Retromolar	0	1 (10%)	0	0	0
Pain	No pain	1 (20%)	1 (10%)	1 (20%)	0	2 (15.4%)
	Painful	4 (80%)	9 (90%)	4 (80%)	4 (100%)	11 (84.6%)
Professional cleaning	Missing	2 (40%)	0	1 (20%)	2 (50%)	4 (30.8%)
	Never	0	2 (22.2%)	1 (20%)	0	3 (23.1%)
	12 months	1 (20%)	1 (11.1%)	0	1 (25%)	1 (7.7%)
	6 months	2 (40%)	6 (66.7%)	3 (60%)	1 (25%)	5 (38.5%)
Tooth brushing	Occasionally	0	1 (11.1%)	1 (20%)	1 (25%)	4 (30.8%)
	1/day	2 (40%)	4 (44.4%)	2 (40%)	0	2 (15.4%)
	2+/day	3 (60%)	4 (44.4%)	2 (40%)	3 (75%)	7 (53.8%)
Smoking habit	Non-smoker	4 (80%)	4 (40%)	3 (60%)	3 (75%)	6 (46.2%)
	Former	1 (20%)	1 (10%)	0	0	2 (15.4%)
	<20 pack-year	0	0	0	0	1 (7.7%)
	>20 pack-year	0	5 (50%)	2 (40%)	1 (25%)	4 (30.8%)
Alcohol	None	3 (60%)	3 (33.3%)	3 (60%)	3 (75%)	4 (30.8%)
	1–5 per month	2 (40%)	2 (22.2%)	0	1 (25%)	4 (30.8%)
	2–7 per week	0	1 (11.1%)	1 (20%)	0	1
	>10 per week	0	3 (33.3%)	1 (20%)	0	4 (30.8%)
Dental Plaque	None	0	1 (11.1%)	0	0	3 (23.1%)
	Minimal	4 (80%)	6 (66.7%)	0	4 (100%)	3 (23.1%)
	Abundant	1 (20%)	2 (22.2%)	5 (100%)	0	7 (53.8%)
Remaining Oral Mucosa	Normal	3 (60%)	3 (33.3%)	1 (20%)	3 (75%)	7 (53.8%)
	Inflamed	1 (20%)	1 (11.1%)	0	0	1 (7.7%)
	Leukoplakia	1 (20%)	2 (22.2%)	2 (40%)	0	2 (15.4%)
	Lichen planus	0	0	0	0	1 (7.7%)
	Polyp	0	1 (11.1%)	1 (20%)	1 (25%)	0
	Nicotine stomatitis	0	0	0	0	1 (7.7%)
	submucous fibrosis	0	1 (11.1%)	0	0	1 (7.7%)
	Melanotic macule	0	1 (11.1%)	0	0	0
	Candidiasis	0	0	1 (20%)	0	0

### Salivary Immune Mediators Are Increased in OSCC Patients

**Figure 2:**
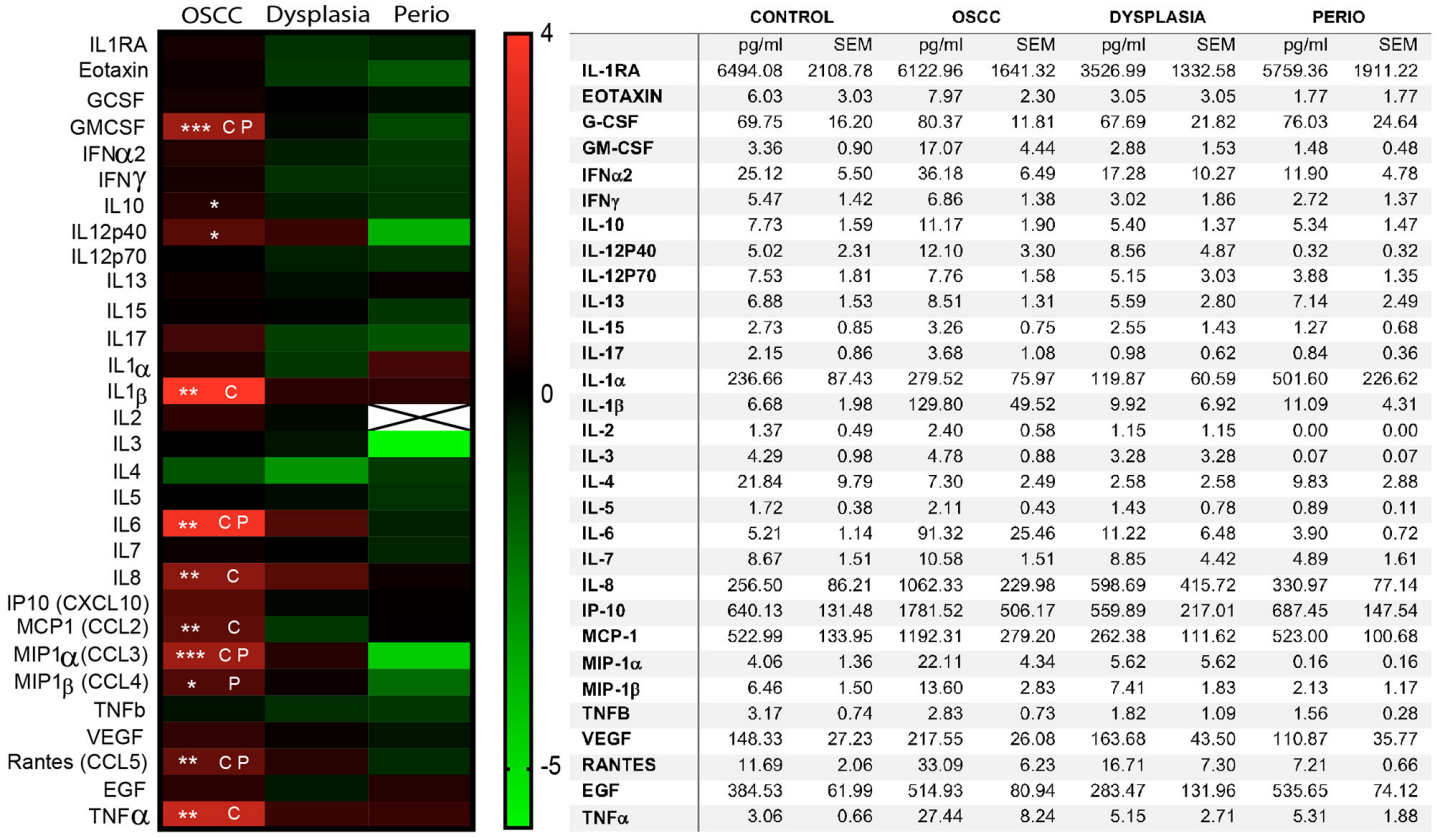
Expression of inflammatory mediators in saliva. The inflammatory mediators were quantified using a 30-plex Luminex panel using non-fixed, filtered saliva. The left panel shows a heatmap of the mean concentration of the individual cytokines compared to control group, using a Log2 scale. The table on the right shows the average concentration of inflammatory mediators in each sample and the standard error of the mean (SEM). (CP) Significant statistical differences between OSCC samples and periodontitis as well as controls. (C) Significant statistical differences between OSCC samples and controls. (P) Significant statistical differences between OSCC and periodontitis samples (*n* = 69, *p < 0.05, **p < 0.01, ***p < 0.001).

Thirty cytokines/chemokine were evaluated in the saliva samples and the results are reported as the fold change of the specific cytokine over control in a log2 scale (Figure 2). There was a significant increase in **GMCSF** (OSCC > Control *p* = 0.0057, OSCC > perio, *p* = 0.0080), **IL-6** (OSCC > control *p* = 0.0001; OSCC > perio *p* = 0.0015), **Rantes/CCL5** (OSCC > control *p* = 0.0079; OSCC > perio *p* = 0.0227) and **MIP1α** (CCL3) (OSCC > control *p* = 0.0034; OSCC > perio *p* = 0.0218) in OSCC patients compared to control and periodontal disease patients. **TNFα** (*p* = 0.0038), **MCP1 (CCL2)** (*p* = 0.0493), **IL-1**β (*P* = 0.0043), and **IL-8** (*p* = 0.0035) were increased in OSCC compared to control only while **MIP1β** (CCL4) (*p* < 0.0365) was increased in OSCC patients compared to periodontal disease patients. **IL-10** (*p* = 0.0364), **IL-12p40** (*p* < 0.0364) were statistically significant but multiple comparison analysis did not show significant differences between groups. There were no significant differences between the dysplasia patients and OSCC patients.

Logistic regression analysis including the eight cytokines increased in cancer compared to control (GMCSF, IL-1β, IL-6, IL-8, Rantes/CCL5, MIP1α, TNFα, MCP1) was performed. The first logistic regression model including IL-6, IL-8, and TNFα showed that elevated IL-6 significantly increased the likelihood of having a diagnosis of cancer (*p* = 0.0040, odds ratio 3.991). Similar analysis including IL-1β, Rantes/CCL5, MCP1, GMCSF, TNFα, and IL-6 showed similar results with elevated IL-6 increasing the probability of having a cancer diagnosis (*p* < 0.0046) (Table 3).

**Table 3:** Models.

		**Analysis of maximum likelihood estimates**				**Odds ratio estimates**			
		**Estimate**	**Standard error**	**Wald (chi square)**	**df**	**Sig**.	**Point estimate**	**95% confidence limits**	
								**Lower**	**Upper**
Model 1	IL6	1.384	0.481	8.279	1	0.004^*^	3.99	1.555	10.242
	TNFa	−0.228	0.433	0.277	1	0.599	0.796	0.341	1.86
	IL8	−0.77	0.38	4.095	1	0.043	0.463	0.22	0.976
	Constant	−1.729	0.544	10.103	1	0.001	0.177		
Model 2	IL6	0.816	0.288	8.034	1	0.005^*^	2.262	1.286	3.978
	IL1β	−0.038	0.073	0.268	1	0.605	0.963	0.835	1.111
	TNFα	−0.273	0.356	0.588	1	0.443	0.761	0.379	1.529
	Constant	−1.662	0.496	11.212	1	0.001	0.19		
Model 3	IL6	0.602	0.245	6.038	1	0.014^*^	1.825	1.13	2.95
	GMCSF	0.236	0.234	1.019	1	0.313	1.266	0.801	2.003
	MCP1	0.059	0.25	0.055	1	0.814	1.061	0.65	1.732
	Constant	−2.046	0.589	12.062	1	0.001	0.129		
Model 4	IL6	0.636	0.261	5.933	1	0.015^*^	1.889	1.132	3.153
	MIP1α	0.509	0.317	2.572	1	0.109	1.663	0.893	3.097
	Rantes(CCL5)	−0.39	0.576	0.459	1	0.498	0.677	0.219	2.093
	Constant	−2.117	0.628	11.362	1	0.001	0.12		

* *Indicates statistically significant*.

### OSCC Patients Have a Distinct Cytokine Profile

**Figure 3:**
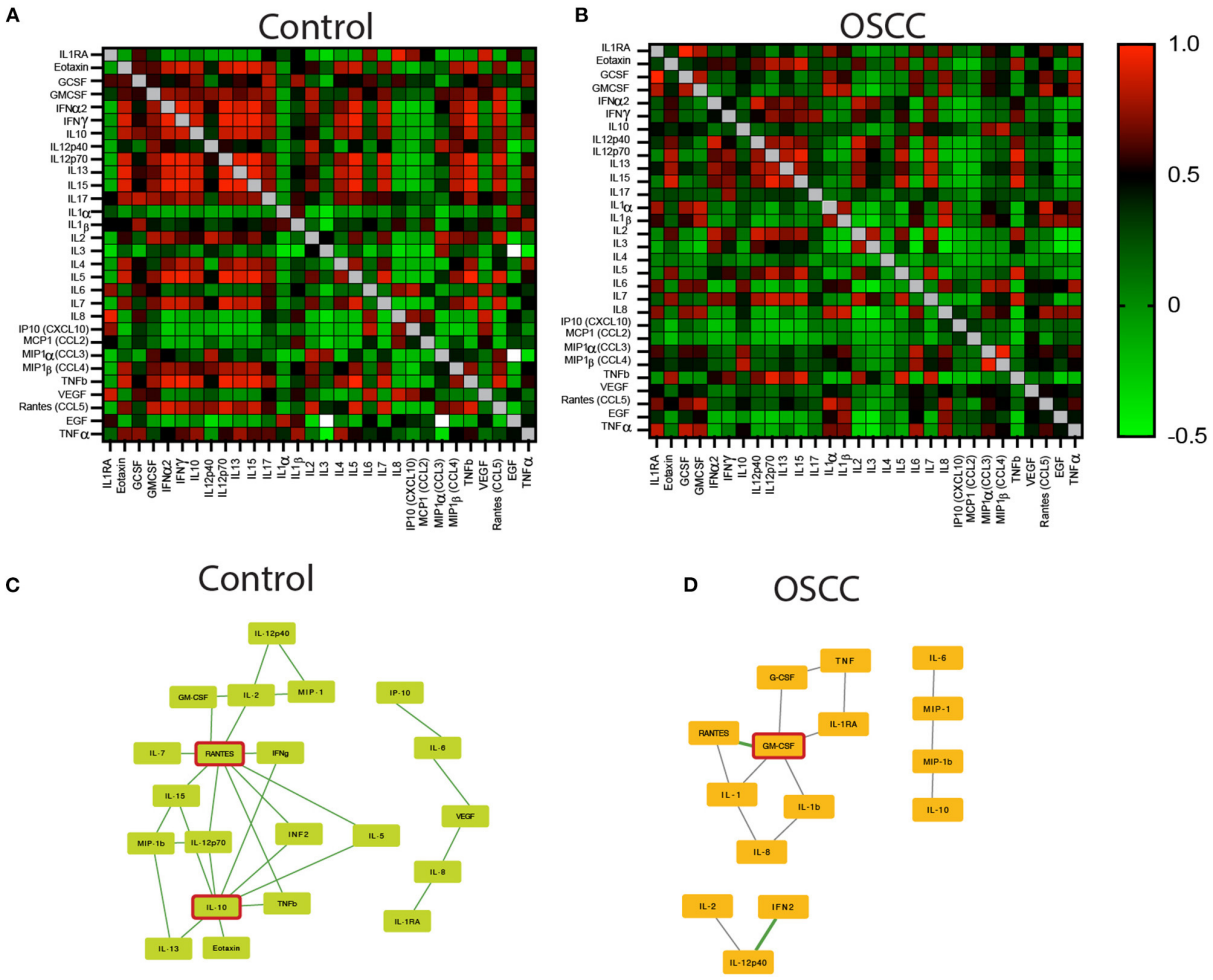
Pearson's correlation analysis between all cytokines in control and OSCC samples. The heatmap represents the r scores (+1 red to −1 green). **(A)** Heatmap of Pearson's correlation of the 30 cytokines in the saliva samples collected from controls. **(B)** Pearson's correlation of the 30 cytokines in the saliva samples of OSCC patients. **(C,D)** A Cytoscape illustrations showing the interaction map of the selected cytokines with statistical differences between controls and OSCC samples. Illustrations are displaying the key role of IL-10 and Rantes/CCL5 in controls **(C)**, and GM-CSF in OSCC samples **(D)** (*n* = 69).

We performed Pearson's correlation analysis between all cytokines in the control and cancer groups (Figures 3A,B). The dysplasia and periodontal disease group were excluded due to the limited number of cases available for analysis. We selected the eleven cytokines that were significantly increased in cancer (GMCSF, IL-6, IL-1β, Rantes/CCL5, MIP1α, MIP1β, TNFα, MCP1/CCL2, IL-8, IL-12p40, IL10) and selected all the positive Pearson's correlations with *r* > 0.8 and significance of *p* < 0.01. The selected cytokine correlations were analyzed using Cytoscape to create an interaction map in control (Figure 3C) and cancer (Figure 3D). The results reveal different networks in each group with IL-10 and Rantes/CCL5 clusters on control samples and GMCSF and IL-6 in cancer.

### Immune Cells in Saliva and Blood

The most common inflammatory cell in saliva was neutrophils (78.61% ± 23.3) followed by lymphocytes (23.98% ± 29.59). The cancer group had the highest number of immune cells (CD45+) in saliva (99,240 ± 38,789) and was significantly higher than control patients (7,945 ± 5,790, *P* < 0.038) but not periodontal disease patients (83,180 ± 21,160). We also calculated the total number of identified cells (CD66b+, CD4+, CD8+, CD56+, CD68+, Siglec8+, CD138+) and OSCC patients showed significantly more cells (55,966 ± 22,151) compared to periodontal disease (32,550 ± 11,816) and control patients (6,355 ± 4,733). Cancer patients also had more neutrophils (*p* = 0.04) and lymphocytes (*p* = 0.05) compared to control patients. There were very few immune cells other than neutrophils and T lymphocytes in saliva, preventing further characterization of the cells (Table 4). The dysplasia group was not included in the total cell count analysis as only two samples were fully processed for cell counts. The differences between the individual cell subtypes were not statistically significant when normalized to the total number of cells identified except for decreased T lymphocytes in periodontal disease group compared to control and dysplasia (Table 5). There were no significant differences in the number of neutrophils according to the clinical appearance of the lesion, presence of plaque or tumor Stage (Supplemental Table 2).

**Table 4:** Distribution of salivary immune cells (numbers).

	**1-Control**			**2-Perio**			**3-Cancer**			**Sig**.	
**Number of cells**	**Mean**	**SEM**	***N***	**Mean**	**SEM**	***N***	**Mean**	**SEM**	***N***	***p***	**pairwise**
Total CD45+	7,945	5,790	13	83,180	31,160	7	99,240	38,780	10	0.0049	3 > 1 (0.032)
Total cells identified (CD66b+, CD4+, CD8+, CD56+, CD68+, Siglec8+, CD138+)	6,355	4,733	13	32,550	11,816	7	55,966	22,151	10	0.0169	3 > 1 (0.041)
Neutrophils (CD45+CD66b+)	6,183	4,677	13	24,963	10,182	7	51,564	22,024	10	0.0099	3 > 1 (0.0384)
Lymphocytes (CD45+CD3+)	464	272	13	268	127	7	3,299	1,552	10	0.0073	3 > 1 (0.0536)
T CD3+CD4+	1	1	13	0	0	7	16	5	10		^***^
T CD3+CD8+	31	21	13	6	3	7	93	32	10		^***^
T CD3+CD25+	23	11	13	22	10	7	92	54	10		^***^
T CD3+PD1+	8	1	8	39	21	7	35	14	8		^***^
T CD3+PDL1+	3	1	8	2	1	7	35	7	8		^***^
Macrophpages (CD45+CD68+)	5	2	13	55	16	7	98	72	10		^***^
Eosinophils (Siglec8+)	16	13	13	7	3	7	35	16	10		^***^
Plasma cells (CD45+CD38+)	4	2	13	17	7	7	116	95	10		^***^
Total PD1+ (CD45+PD1+)	64	8	8	180	25	7	130	11	8		^***^
Total PDL1+ (CD45+PDL1+)	21	8	8	63	25	7	54	11	8		^***^

*The results are based on the total count of positive cells by Flow cytometry. Statistical analyses between groups were performed using independent samples Kruskall Wallis test and Bonferroni correction*.

*** *Group comparisons were not calculated due to the very limited number of cells detected (<200 cells per sample) in the saliva samples*.

**Table 5:** Distribution of salivary immune cells (percentage of all identified cells).

**Percent of total**	**1-Control**			**2-Perio**			**3-Cancer**			**4-Dysplasia**			**Sig**.
	**Mean**	**SEM**	**N**	**Mean**	**SEM**	**N**	**Mean**	**SEM**	**N**	**Mean**	**SEM**	**N**	
Neutrophils (CD45+CD66b+)	0.76	0.056	23	0.757	0.076	8	0.814	0.037	31	0.768	0.141	5	
Lymphocytes (CD45+CD3+)	0.311	0.056	23	0.031	0.021	8	0.169	0.039	31	0.288	0.1	5	1 AND 4 >2
													(0.001, 0.047)
T CD3+CD4+	0.005	0.001	23	0.001	0.001	8	0.019	0.009	31	0.019	0.012	5	
T CD3+CD8+	0.17	0.045	23	0.066	0.037	8	0.088	0.021	31	0.063	0.016	5	
T CD3+CD25+	0.216	0.056	23	0.092	0.027	8	0.15	0.032	31	0.208	0.098	5	
TCD3+PD1+	0.199	0.054	23	0.115	0.035	8	0.164	0.037	31	0.042	0.023	5	
TCD3+PDL1+	0.115	0.036	23	0.023	0.017	8	0.043	0.015	31	0.02	0.015	5	
Macrophages (CD45+CD68+)	0.012	0.005	23	0.014	0.012	8	0.038	0.016	31	0.067	0.057	5	
Eosinophils (Siglec8+)	0.034	0.031	23	0	0	8	0.012	0.005	31	0.048	0.039	5	
Plasma cells (CD45+CD38+)	0.008	0.005	23	0	0	8	0.006	0.002	31	0.01	0.008	5	
Total PD1+ (CD45+PD1+)	0.029	0.011	23	0.012	0.009	8	0.003	0.001	31	0.001	0.001	5	
Total PDL1+ (CD45+PDL1+)	0.022	0.006	23	0.002	0.001	8	0.004	0.002	31	0.001	0.001	5	

*The percentage of the cells was calculated as the number of individual cell subtypes divided by the number of all cells identified (CD66b+, CD4+, CD8+, CD56+, CD68+, Siglec8+, CD138+)*.

We have analyzed complete blood counts from all cancer patients and found no significant difference in complete blood counts between stages of disease. There was no correlation between the salivary cell counts and blood counts for each patient and all the differential blood cell counts in OSCC were within normal limits (data not shown).

### Tissue Analysis

**Figure 4:**
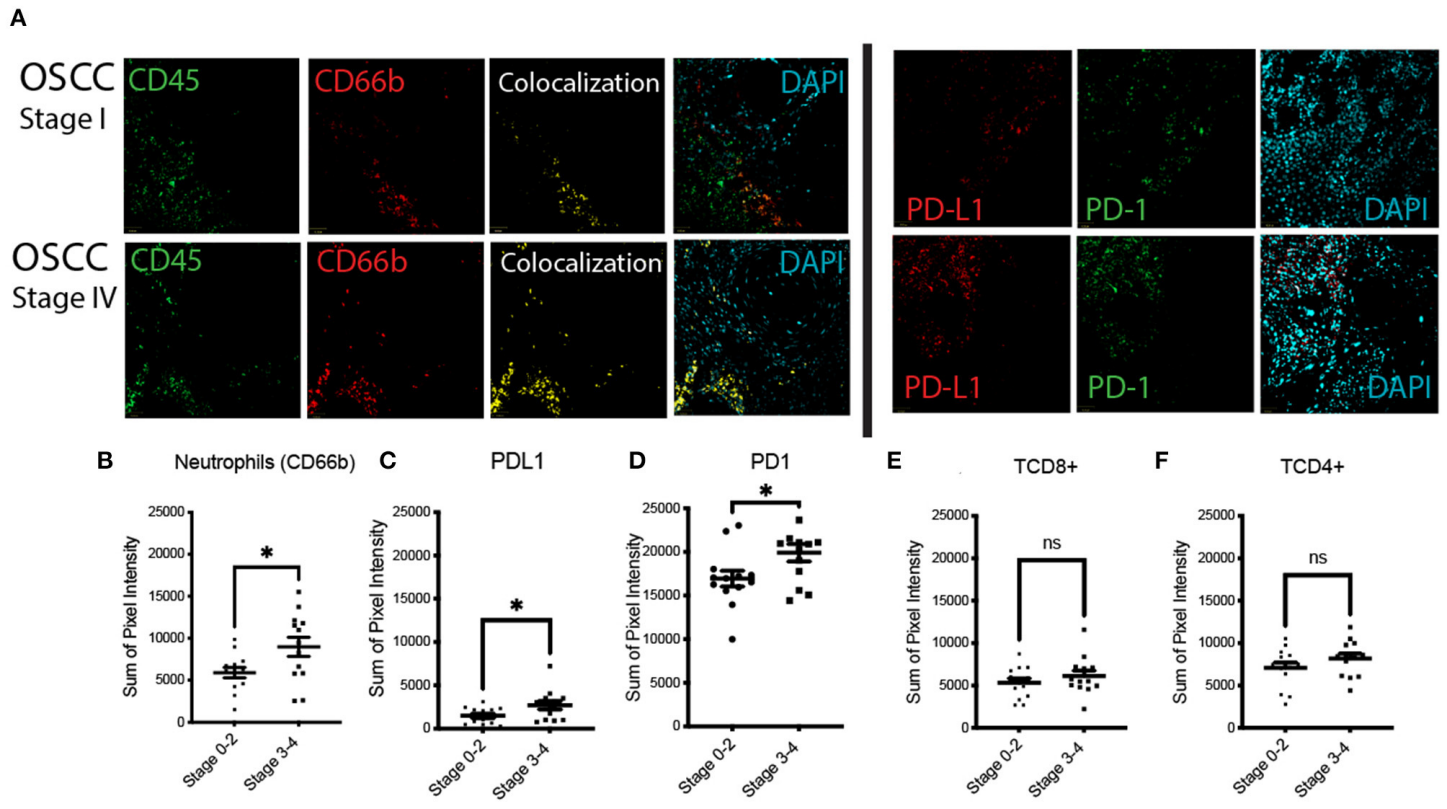
Expression of CD45, CD66b (neutrophils), PD-1/PD-L1 in low stage (Stage I and II), and high stage (Stage III and IV) OSCC. **(A)** Fluorescent immunohistochemistry (FIHC) stained samples showing aPD-1/PD-L1 and neutrophils (colocalized CD45 and CD66b) in stage IV OSCC samples compared to stage I. **(B–D)** Quantification of neutrophils **(B)**, PD-L1 **(C)**, and PD-1 **(D)** positive cells in high Stage (III–IV) tumors compared to low stage (0–II) tumors. **(E,F)** Graphs showing no significant differences in T cells between different tumor stages. (*n* = 25, *p < 0.05).

We investigated neutrophils (CD45+ CD66b+), CD8+ T cells (CD3+CD8+), and CD4+ T cells (CD3+CD4+) in addition to PD-1 and PD-L1 expression in 25 tissue samples that were available from the cancer cohort (Figure 4A). To normalize the number of samples in each group, we combined Stage 0–2 (low stage) and Stage 3–4 (high stage) patients. There was an increase in neutrophils (CD45+CD66b+) (*P* = 0.0262) in high Stage (III–IV) tumors compared to low stage (0-II) tumors (Figure 4B). There was also more PD-L1+ (*P* = 0.038) (Figure 4C) and CD45+ PD-1+ (*P* = 0.038) (Figure 4D) cells in high stage patients. No significant differences were found between the other cells tested including CD3+CD4+ (*P* = 0.219) and CD3+CD8+ (*p* = 0.33) T cells (Figures 4E,F).

### Factors Associated With Survival

**Figure 5:**
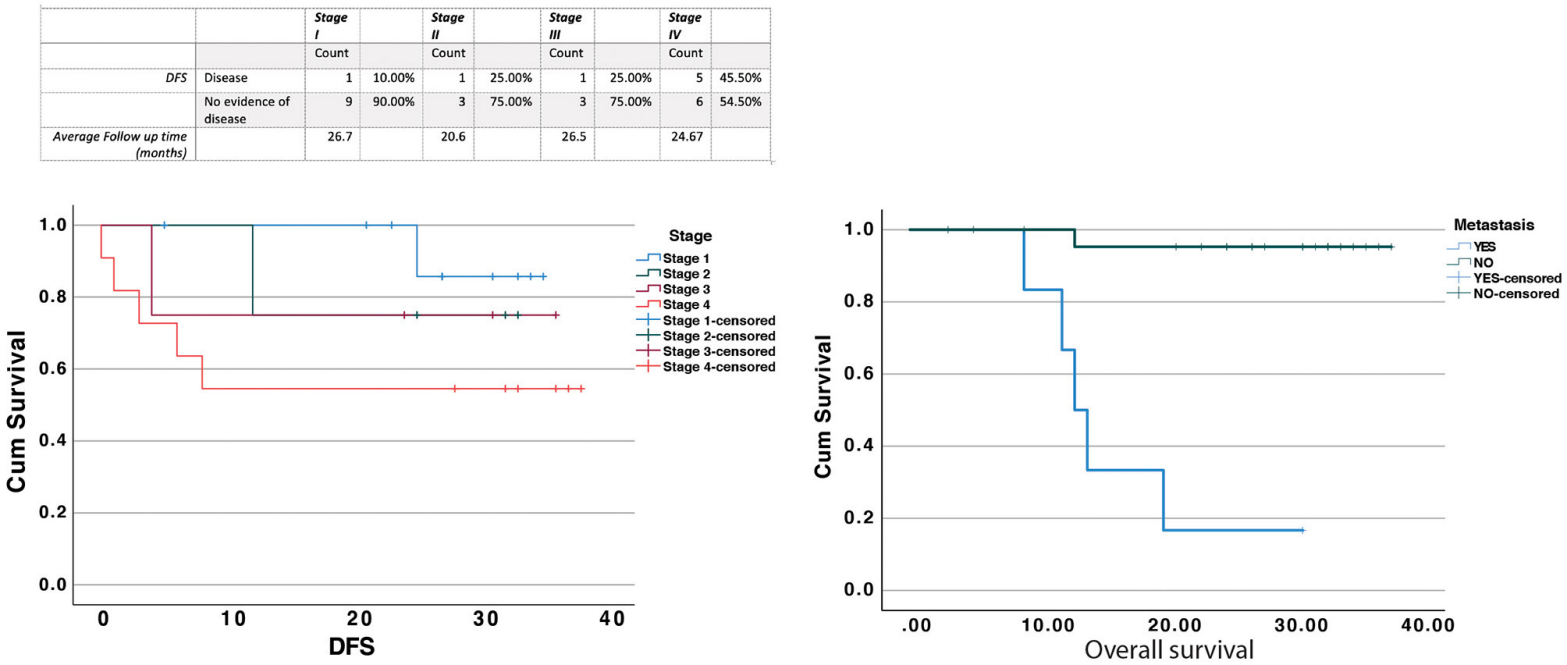
Patient outcomes were determined based on the review of the electronic patient records at Sunnybrook hospital. Patients that had no follow up after diagnosis were considered as lost to follow up and were not included in the table above. The results were analyzed using the Log Rank test for Stage (Log Rank test, *p* = 0.309) and presence of metastasis (Log Rank test, *p* = 0.0001).

The patient outcomes are summarized on Figure 5. We had up to 35 months for follow-up information and from the original cohort of cancer patients. Excluding dysplasia patients, eight patients had recurrences or died of disease (8/32, 25%). Stage IV patients had the worst outcome with 33.3% dying of disease at the end of the follow up period. Bivariate Cox models and Kaplan Meier analyses were used to determine potential predictors of diseased free survival (DFS). Age, gender, location, cytokines, and salivary immune cells were tested but no variables achieved statistical significance. Figure 5 shows Kaplan Meier curves for Stage (Log Rank test, *p* = 0.309) and presence of metastasis (Log Rank test, *p* = 0.0001).

## Discussion

Understanding the characteristics of the inflammatory infiltrate in oral cancer is essential to determine pathogenesis and new strategies to predict outcomes and control the disease. In this pilot study, we have created and tested a protocol to collect saliva samples for a comprehensive analysis of inflammatory cells and mediators and used it to study the inflammatory signature of oral cancer patients compared to control and periodontal disease patients. Our protocol is based on a single collection that can identify 30 cytokines and was also able to identify cell surface markers in salivary immune cells. This pilot study allowed us to optimize a non-invasive collection and analysis and the results will be used to create a large clinical study to characterize the salivary inflammatory signature of oral cancer patients. The protocol also allows for repeated sampling.

### Developing a Protocol for Collecting and Analysis

The analysis of inflammatory cell profile in saliva by FCM is challenging, and we believe this is the first report of its kind. Saliva inherently contains debris and proteases that contribute to sample degradation and formation of aggregates. To minimize this, we used multiple filtrations prior to processing the samples. The use of formalin has the potential to alter antigen availability, therefore, compensation was done using fixed saliva from the same donor and the antibodies were tested in both fixed and fresh saliva. To determine the different cell populations and exclude debris, gating was carefully done using forward and side scatter and by applying multiple markers/gates for each cell. An important limitation of studying inflammatory cell profile in saliva by FCM is the number of cells available in each sample, particularly in control patients. By vigorously rinsing with saline, we were able to collect at least 100,000 inflammatory cells per sample which made FCM analysis feasible. To achieve reproducible results, we had to have access to filters, fixatives, protease inhibitors, ice, and a centrifuge adjacent to the collection area. This allowed us to minimize the time between collection, processing, and storage which was critical to achieve consistency.

### Unique Characteristics of Salivary Immune Cells and Mediators

Our results show an increased number of inflammatory cells and a distinct change in inflammatory mediators in the saliva of cancer and periodontal disease patients compared to control. The levels of salivary inflammatory mediators GMCSF, IL-6, Rantes/CCL5, MIP1α (CCL3), and MIP1β (CCL4) were elevated in OSCC compared to periodontal disease which supports that the inflammatory response in OSCC is specific and different from other causes of chronic inflammation such as periodontitis in the oral cavity. The changes in immune cells in saliva of OSCC patients were primarily due to an increase in total number of cells and not from specific shifts in different cell populations. This is potentially due to the fact the highly motile neutrophils would extravasate to saliva while most other inflammatory cells would stay attached to the stroma or connective tissue, therefore the changes in immune cells are expected to be seen in the tissue and not in saliva. The findings also raise the possibility that neutrophils in saliva are primarily caused by the presence of ulceration rather than a specific recruitment mechanism. Several findings challenge this hypothesis. First, there was no difference in the number of salivary neutrophils in lesions that were primarily ulcerated vs. non-homogeneous hyperkeratotic lesions or exophytic lesion. Second, there were no significant differences between the number of salivary neutrophils in different cancer Stages, suggesting that tumor size alone does not explain the observed neutrophilia. Finally, some patients with non-ulcerated lesions in periodontal disease showed similar levels of salivary neutrophils compared to cancer patients, suggesting that ulceration alone also does not explain the increase recruitment of neutrophils. Our data supports the conclusion that the increased number of immune cells in saliva of OSCC patients is due to the recruitment of inflammatory cells to the tumor microenvironment [11] which thus leads to more immune cells that come into direct contact with saliva. Further studies are needed to determine if other inflammatory, reactive, and ulcerated oral lesions also lead to salivary neutrophilia.

### Pro-inflammatory Pathways Activated in Cancer

Our results suggest a markedly pro-inflammatory immune environment in OSCC. Signaling pathways downstream of growth factors and cytokines such as interleukin IL-1β, IL-6, TNFα, vascular endothelial growth factor (VEGF), and epidermal growth factor (EGF) [22, 25, 26] are complex and commonly rely on Nuclear factor kappa beta (NF-kB), and to a lesser extent signal transducer and activator of transcription 3 (STAT3). These pathways are also critical in carcinogenesis in head and neck cancer and may link the immune landscape of OSCC to tumor promotion [22, 27, 28]. Nuclear factor kappa beta and STAT3 hyperactivation in tumor cells and immune cells in the tumor microenvironment in turn promotes production of several pro-inflammatory cytokines including IL-1β, IL-6, and TNFα, which promote survival and proliferation of tumor cells [22, 24, 27]. In addition, both STAT3 and NF-kB interfere with p53 synthesis, and attenuate p53-mediated genomic surveillance [27]. This is also supported by our recent results that show that neutrophils and TNFα promotes the invasion of oral cancer cells *in vitro* [11]. Previous studies showed that an increased neutrophilic infiltration in OSCC tissue is associated with poor survival in advanced HNSCC [29–31]. Specifically, Wang et al. showed that tongue SCC associated with neutrophils infiltration displayed higher clinical stage, increased lymph node metastasis, and increased chance of tumor recurrence [29]. Our results support this pathway as higher stage OSCC showed more neutrophils. The pro-inflammatory response is in contrast with an increase in PD-1 and PDL-1 in high stage OSCC as this might be a specific immunomodulatory response from the tumor cells.

## Conclusion

This pilot study shows that salivary and tissue analysis of a broad panel of inflammatory mediators and cells is feasible and can be used to characterize in detail the immune responses that are unique to OSCC. Despite the small sample size, our results indicate that OSCC patients show an increase in immune cells in the tumor microenvironment and a unique salivary cytokine profile. These findings can be used to design clinical tests that could be used as part of treatment decision making; such assays can help to identify patients at higher risk of developing disease adverse events and recurrence. For example, OSCC patients with elevated salivary pro-inflammatory chemokines/cytokines after definitive treatment may indicate higher risk of recurrence or persistent disease activity. This could be incorporated as part of the regular follow up visits. Regarding the development of unique cancer therapies, there are numerous ongoing studies in different cancers targeting chemokine networks and immunomodulatory pathways in cancer treatment as recently reviewed in [32, 33]. Therefore, our results will be used to identify the specific inflammatory pathways involved in the pathogenesis and progression of oral cancer and enable the development of pre-clinical studies and clinical trials using targeted immunotherapy.

Saliva is easy and quick to collect as per our protocol, making it a potential non-invasive, easy to perform, saliva-based test. Using our collection protocol, saliva collection can be performed in a variety of clinical settings by various health professionals, regardless of clinical expertise for assessing presence of oral cancer. Lastly, in the era of immunotherapy, finding biomarkers to identify cancer patients eligible for immunotherapy is urgent. In this study, we set the basis on which we can build to further explore the characteristics of inflammatory mediators in the saliva and their value as predictive markers.

## Data Availability Statement

The raw data supporting the conclusions of this article will be made available by the authors, without undue reservation.

## Ethics Statement

The studies involving human participants were reviewed and approved by the Research Ethics Board (REB) of Sunnybrook Health Sciences Center (project identification number 223-2015) and the University of Toronto (protocol number 32724). The patients/participants provided their written informed consent to participate in this study.

## Author Contributions

CL completed all patient recruitment and data collection and helped in the data analysis. MM has conceptualized the project, analyzed data, and wrote the manuscript. NN has completed all the tissue analysis experiments. AK completed all statistical analyses. KH has provided clinical support to recruit patients.GB participated in the creation of the project and manuscript review. DE and AA have provided technical support and development. All authors contributed to the article and approved the submitted version.

## Funding

This study was funded by the University of Toronto, Faculty of Dentistry, Oral Pathology Department research fund.

## Conflict of Interest

The authors declare that the research was conducted in the absence of any commercial or financial relationships that could be construed as a potential conflict of interest.

## Publisher's Note

All claims expressed in this article are solely those of the authors and do not necessarily represent those of their affiliated organizations, or those of the publisher, the editors and the reviewers. Any product that may be evaluated in this article, or claim that may be made by its manufacturer, is not guaranteed or endorsed by the publisher.
