# Supplementary material for: Characterization of Oral Squamous Cell Carcinoma Associated Inflammation: A Pilot Study
Source: Front Oral Health. 2021 Sep 21;2:740469. doi: 10.3389/froh.2021.740469 (PMC8757876; doi:10.3389/froh.2021.740469)
Supplement: Supplementary file 1 [file Table_1.docx]

| Laser | Detector | Target antigen | Inflammatory cell | Fluorophore | Clone | Supplier |
| --- | --- | --- | --- | --- | --- | --- |
| Blue 488 nm | 530/30 | CD3 | T lymphocytes | FITC | HIT3a | BioLegend |
| Blue 488 nm | 710/50 | CD56 | NK cells/NKT cells | PerCP-eFluor 710 | CMSSB | eBiosciences |
| Red 640 nm | 670/30 | Siglec8 | Eosinophils | APC |  | BioLegend |
| Red 640 nm | 780/60 | CD8 | CD8+ T lymphocyte | APC/Cy7 |  | BioLegend |
| Violet 405 nm | 450/50 | CD66b | Neutrophils | BV421 | G10FS | BD Biosciences |
| Violet 405 nm | 610/20 | CD279 | PD1 | BV605 | EH12.2H7 | Biolegend |
| Violet 405 nm | 780/60 | CD274 | PDL1 | BV785 | 29E.2A3 | Biolegend |
| Yellow/Green 561 nm | 586/15 | CD68 | Macrophages | PE | Y1/82A | BioLegend |
| Yellow/Green 561 nm | 610/20 | CD25 | Activated lymphocytes | PE-CF594 | M-A251 | BD Biosciences |
| Yellow/Green 561 nm | 780/60 | CD138 | Plasma cells | PE/Cy7 | MI15 | BioLegend |
| UV | 450/50 | CD45 | Leukocytes | BUV805 | HI30 | BD Horizon |
| UV | 379/29 | CD4 | CD4+ T lymphocytes | BUV395 | SK3 | BD Biosciences |

Supplemental table 1: Details of lasers, detectors and antibodies used in the flow cytometry experiments
