# Supplementary material for: Characterization of Oral Squamous Cell Carcinoma Associated Inflammation: A Pilot Study
Source: Front Oral Health. 2021 Sep 21;2:740469. doi: 10.3389/froh.2021.740469 (PMC8757876; doi:10.3389/froh.2021.740469)
Supplement: Supplementary file 2 [file Table_2.docx]

|  |  | Neutrophils | | Neutrophils, % | |
| --- | --- | --- | --- | --- | --- |
|  |  | Mean | SEM | Mean | SEM |
| Dental Plaque  (N.S.) | (None, minimal) | 33244 | 24639 | 0.777 | 0.055 |
|  | (Moderate-abundant) | 39605 | 15947 | 0.824 | 0.038 |
| Clinical Stage  (N.S.) | Low Stage (I, II) | 49980 | 34321 | 0.806 | 0.056 |
|  | High Stage (III, IV) | 53149 | 31679 | 0.822 | 0.05 |
| Clinical Appearance  (N.S.) | Ulcerated | 38347 | 34950 | 0.883 | 0.039 |
|  | Non-homogenous leukoplakia | 73700 | 36571 | 0.731 | 0.072 |
|  | Exophytic,  Tumefaction | 14604 | 11378 | 0.854 | . |

Supplemental Table 2: Number and percentage of neutrophils in saliva according to clinical appearance, clinical Stage and presence of plaque. Standard error of the mean (SEM), Not significant, N.S., P>0.05
